# Potential therapeutic strategies for MASH: from preclinical to clinical development

**DOI:** 10.1093/lifemeta/loae029

**Published:** 2024-07-06

**Authors:** Zhifu Xie, Yufeng Li, Long Cheng, Yidan Huang, Wanglin Rao, Honglu Shi, Jingya Li

**Affiliations:** State Key Laboratory of Drug Research, Shanghai Institute of Materia Medica, Chinese Academy of Sciences, Shanghai 201203, China; State Key Laboratory of Drug Research, Shanghai Institute of Materia Medica, Chinese Academy of Sciences, Shanghai 201203, China; State Key Laboratory of Drug Research, Shanghai Institute of Materia Medica, Chinese Academy of Sciences, Shanghai 201203, China; University of Chinese Academy of Sciences, Beijing 100049, China; State Key Laboratory of Drug Research, Shanghai Institute of Materia Medica, Chinese Academy of Sciences, Shanghai 201203, China; University of Chinese Academy of Sciences, Beijing 100049, China; State Key Laboratory of Drug Research, Shanghai Institute of Materia Medica, Chinese Academy of Sciences, Shanghai 201203, China; University of Chinese Academy of Sciences, Beijing 100049, China; School of Pharmaceutical Science and Technology, Hangzhou Institute for Advanced Study, University of Chinese Academy of Sciences, Hangzhou, Zhejiang 310024, China; State Key Laboratory of Drug Research, Shanghai Institute of Materia Medica, Chinese Academy of Sciences, Shanghai 201203, China; State Key Laboratory of Drug Research, Shanghai Institute of Materia Medica, Chinese Academy of Sciences, Shanghai 201203, China; University of Chinese Academy of Sciences, Beijing 100049, China; School of Pharmaceutical Science and Technology, Hangzhou Institute for Advanced Study, University of Chinese Academy of Sciences, Hangzhou, Zhejiang 310024, China

**Keywords:** steatosis, inflammation, fibrosis, metabolic dysfunction-associated steatohepatitis, pharmacotherapy

## Abstract

Current treatment paradigms for metabolic dysfunction-associated steatohepatitis (MASH) are based primarily on dietary restrictions and the use of existing drugs, including anti-diabetic and anti-obesity medications. Given the limited number of approved drugs specifically for MASH, recent efforts have focused on promising strategies that specifically target hepatic lipid metabolism, inflammation, fibrosis, or a combination of these processes. In this review, we examined the pathophysiology underlying the development of MASH in relation to recent advances in effective MASH therapy. Particularly, we analyzed the effects of lipogenesis inhibitors, nuclear receptor agonists, glucagon-like peptide-1 (GLP-1) receptor (GLP-1R) agonists, fibroblast growth factor mimetics, and combinatorial therapeutic approaches. We summarize these targets along with their preclinical and clinical candidates with the ultimate goal of optimizing the therapeutic prospects for MASH.

## Introduction

Nonalcoholic fatty liver disease (NAFLD) is a major chronic liver disease characterized by excess lipid accumulation, inflammation, and hepatocyte injury [1–3]. Notably, the incidence of NAFLD is increasing, and this disease is estimated to affect approximately 25% of the adult population worldwide. This pattern is consistently associated with global increases in metabolic syndrome, obesity, and diabetes [4]. The global burden of NAFLD is projected to increase two to three times by 2030 [5, 6]. For example, in the USA, the number of NAFLD cases is expected to reach 100 million by 2030, up from 80 million in 2015 [7, 8]. Similarly, China experienced a sharp increase in NAFLD incidence in a short period from 2008 to 2018, with the highest number of patients worldwide and a prevalence of 29.2% [9]. As a result, a significant proportion of simple steatosis progresses to nonalcoholic steatohepatitis (NASH), a more severe form of NAFLD. Recently, metabolic dysfunction-associated steatotic liver disease (MASLD), which has been described as the hepatic manifestation of metabolic syndrome and a continuum from obesity to a series of metabolic disorders, was suggested as a more appropriate overarching term for NAFLD [10].

Metabolic dysfunction-associated steatohepatitis (MASH), formerly known as NASH, is characterized by excess lipid accumulation (steatosis), inflammation, injury, and fibrosis in the liver and if left uncontrolled, can lead to cirrhosis or liver cancer [8, 11]. Steatosis accounts for the virulent nature of MASH and initiates its development. Increased fat in the liver leads to the accumulation of hepatocyte injury inducers (e.g., lipotoxicity), which triggers inflammatory responses and immune cell infiltration into the liver. Liver fibrosis progressively develops through the activation of hepatic stellate cells (HSCs), which are the major sites of fibrogenesis [12]. Notably, MASH is the fastest growing cause of hepatocellular carcinoma (HCC) in liver transplant candidates and age-adjusted liver cancer deaths worldwide [8]. Therefore, immediate action and increased awareness are needed to address the growing prevalence and risks associated with MASH.

In recent years, our understanding of the pathophysiology and management of MASLD has advanced significantly. For patients with simple steatosis, lifestyle interventions such as exercise, dietary changes, and weight loss are the main treatment strategies used to mitigate the progression of MASLD [13, 14]. However, lifestyle adjustments alone are not enough to reverse more advanced MASLD with severe inflammation and fibrosis. Therefore, the development and use of drug therapies in combination with lifestyle interventions are essential. To date, the groundbreaking approval of the thyroid hormone receptor beta (THRβ) agonist resmetirom (MGL-3196) by the United States Food and Drug Administration (FDA) in 2024 represents the first-ever selective treatment for MASH. Currently, numerous clinical trials are underway, raising the expectation of more therapeutic breakthroughs [13, 15–18]. This review summarizes MASH treatment strategies in clinical trials and introduces several novel therapies for MASH, thereby contributing to the advancement of knowledge in this critical area.

## The main features and pathological processes of MASH

Insulin resistance, diabetes, obesity, and hyperlipidemia are significant contributors to the pathophysiology of MASH. The “multiple hit” hypothesis accounts for the simultaneous effects of multiple injuries on predisposed individuals and offers a more accurate understanding of the pathogenesis of MASH. These injuries contribute to the three main pathological characteristics of MASH, namely, excessive lipid accumulation (steatosis), immune cell infiltration (inflammation), and fibrosis caused by HSC activation.

### Excessive lipid accumulation induces steatosis and lipotoxicity

The liver plays a unique role in lipid metabolism and maintains lipid concentrations at normal levels [19]. Fat accumulation in the liver occurs through various mechanisms, including adipose tissue lipolysis, *de novo* lipogenesis (DNL), and dietary fat absorption [20]. In insulin resistance, excessive activation of lipolysis in adipose tissue increases circulating levels of free fatty acids (FFAs), which ultimately promotes fat deposition in the liver and contributes to fatty liver disease [21]. In addition, excess intake of dietary carbohydrates promotes hepatic DNL in the liver [22–24]. Indeed, isotope-labeling studies have shown that liver DNL is significantly upregulated in individuals with MASLD [25]. The contribution of DNL to liver triglyceride (TG) synthesis in the fed state is approximately three to six times greater in the livers of obese MASLD patients than in those of normal individuals [25, 26].

Liver DNL is driven by increased consumption of acarbohydrate-rich diet (e.g., fructose), or in part, by dysregulated transcriptional regulation of hepatic lipogenesis under conditions of insulin resistance [23]. Two important transcription factors regulate the enzymes that catalyze lipogenesis in the liver: sterol regulatory element-binding protein 1c (SREBP1c) and carbohydrate regulatory element-binding protein (ChREBP). SREBP1c-mediated target gene transcription is activated by insulin stimulation, while ChREBP is activated by carbohydrate metabolites, which accumulate in the livers of individuals with MASLD [24]. SREBP1c and ChREBP increase the expression of several lipogenic genes, including ketohexokinase (*KHK*), acetyl coenzyme A (acetyl-CoA) carboxylase (*ACC*), fatty acid synthase (*FASN*), and stearoyl-CoA desaturase 1 (*SCD-1*). In parallel with lipogenesis, defects in the β-oxidation of mitochondrial fatty acids (FAs) in the liver also contribute to the development of hepatic steatosis and MASH progression [27, 28]. It is crucial to emphasize the important role of cholesterol and cholesterol esters as essential factors in the progression from steatosis to MASH. Excess cholesterol can significantly exacerbate cellular toxicity as well as proinflammatory and profibrotic effects in hepatocytes, immune cells, and HSCs [29]. Inhibiting cholesterol biogenesis and absorption or increasing cholesterol efflux is expected to attenuate MASH [30–33]. THRβ agonists, which facilitate FA degradation and cholesterol biosynthesis, collectively exert remarkable therapeutic effects that lead to improvements in MASH [34, 35].

Lipotoxicity may be accompanied by organelle dysfunction, cellular apoptosis, or necrosis, and is closely related to chronic inflammation. Saturated FFAs, such as palmitate (C16:0) and stearate (C18:0), exert direct cytotoxic effects. Furthermore, sphingolipid levels are increased in MASH [36, 37], and ceramide has recently been linked to the pathophysiology of this disease [38]. Hepatocyte apoptosis is significantly increased in patients and animals with MASH, is positively correlated with disease severity, and is considered one of the pathologic hallmarks of MASH [39]. Apoptotic caspase deficiency (e.g., caspase-8 and caspase-3) has been reported to protect against liver injury and fibrosis in murine models of MASH [40, 41]. Consequently, the development of drugs against lipotoxicity and cell death in MASLD has recently attracted increased attention.

### Immune cell infiltration accelerates the process of MASH

Excess lipids disrupt the normal function of hepatocytes and trigger endoplasmic reticulum (ER) stress, mitochondrial dysfunction, and production of reactive oxygen species (ROS) [15]. These events activate critical signaling pathways, including the c-Jun N-terminal kinase (JNK) pathway and nuclear factor-kappa light chain enhancer of activated B cells (NF-κB) pathway, thereby regulating gene expression to induce inflammation and apoptosis, which are the main drivers of MASH progression [42, 43].

In livers, Kupffer cells (KCs) are the main source of inflammatory cytokines. KCs sense intestinal bacterial products and endogenous substances released by damaged cells via Toll-like receptors (TLR4, TLR2, and TLR9) and nuclear nucleotide-binding oligomerization domain (NOD)-like receptors (NLRs). This recognition triggers the nuclear translocation of NF-κB and the production of various proinflammatory and profibrotic cytokines, including tumor necrosis factor-alpha (TNFα), interleukin-1beta (IL-1β), C-C motif chemokine ligand 5 (CCL5), and transforming growth factor-beta (TGF-β) [44, 45]. Neutrophil infiltration from the circulation is closely associated with MASLD/MASH, which involves abnormal inflammatory leukocytes. Furthermore, adaptive immunity also promotes MASLD/MASH development, which is characterized by increased T helper type 17 (Th17) cells and IL-17A expression [46, 47]. Additionally, the inhibition of C-C motif chemokine receptors (CCRs) is a common therapeutic approach. Based on these findings, novel therapeutics targeting alternative proinflammatory pathways, such as those involving IL-27, IL-17, IL-11, IL-1, and TNFα, have emerged as promising candidates for the treatment of MASLD/MASH [18, 48]. Targeting self-aggressive CD8^+^ T cells and antiplatelet therapy are also potential strategies for alleviating MASLD or preventing its progression to HCC [49].

### HSC activation promotes the development of fibrosis

MASLD can progress from MASH with mild symptoms to irreversible liver fibrosis and even liver cancer. Therefore, effective intervention for liver fibrosis is the most important step to prevent the progression of MASH to cirrhosis and HCC [50, 51]. HSCs are central to liver fibrosis, and their subsets either regulate immune mechanisms through chemokines and cytokines or differentiate into matrix-producing myofibroblasts.

Among several growth factors that play key roles in the development of fibrosis induced by HSCs, TGF-β is the most potent profibrotic cytokine. TGF-β is secreted in a latent form by various hepatocyte populations and is partially activated by HSCs that express integrin αv [52]. The inhibition of TGF-β activation by targeting cell surface integrin subpopulations that contain αv (e.g., integrins αvβ1, αvβ3, αvβ5, αvβ6, and αvβ8) is considered a promising strategy [53, 54]. Another cytokine that promotes fibrosis is platelet-derived growth factor (PDGF). HSCs express high levels of PDGF receptors, and activation of HSCs can induce their proliferation and migration, thus enhancing their role in liver fibrosis. Consistent with this mechanism, multikinase inhibitors that specifically target PDGF and its isoforms have shown the ability to counteract liver fibrosis [55]. Furthermore, vascular endothelial growth factor (VEGF) produced by hepatocytes induces the activation and proliferation of HSCs, which leads to increased production of extracellular matrix (ECM) proteins and TGF-β, thereby inducing and exacerbating liver fibrosis [56]. These results suggest that inhibition of HSC activation by cytokine stimulation may be an effective approach to combat liver fibrosis.

In addition to activation of HSCs by cytokines and other factors, immune cell-mediated killing of hepatocytes also contributes to liver fibrosis. Profibrotic inflammatory chemokines and their receptors, such as CCL2, CCL21, IL-8, IL-17, IL-22, C-X-C motif chemokine ligand 9 (CXCL9), CXCL10, CXCL11, and C-X-C motif chemokine receptor 1 (CXCR1), are involved in this process [57]. As sustained activation of immune response further aggravates liver fibrosis, inhibition of specific chemokines and NF-κB can attenuate the progression of liver fibrosis [58].

## Advancements in the development of innovative anti-MASH drugs

**Figure 1:**
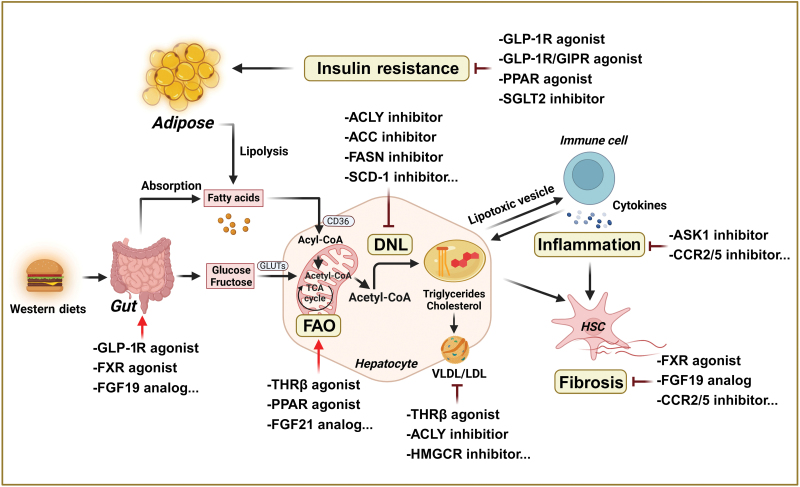
Potential therapeutic strategies for MASH. Multiple pathways and a wide range of pathophysiological processes contribute to MASH. The three most common features of MASH are excess lipid accumulation, inflammation, and fibrosis. Modifications in lifestyle and the use of currently prescribed drugs, such as insulin resistance agents, are beneficial in the management of MASH. Novel strategies that specifically target the liver for the treatment of inflammation, fibrosis, and hepatic lipid metabolism are being investigated with promising outcomes at various stages of preclinical and clinical studies. DNL, *de novo* lipogenesis; FAO: fatty acid oxidation; ACLY: ATP citrate lyase; ACC: acetyl coenzyme A (acetyl-CoA) carboxylase; FASN, fatty acid synthetase; SCD-1: stearoyl-CoA desaturase-1; HMGCR: 3-hydroxy-3-methylglutaryl-CoA reductase; THRβ: thyroid hormone receptor β; PPAR: peroxisome proliferator-activated receptor; FXR: farnesoid X receptor; ASK1: apoptosis signal-regulating kinase 1; GLP-1R/GIPR: glucagon-like peptide-1 receptor/gastric inhibitory polypeptide receptor; SGLT2: sodium-glucose cotransporter-2; CCR2/5: C–C motif chemokine receptor 2/5; VLDL/LDL: very low-density lipoprotein/low-density lipoprotein.

Lipid accumulation, inflammation, and fibrosis are the primary pathological features of MASH. Based on extensive research of these pathological mechanisms, the development of innovative therapeutics for MASH is promising. However, the need for pathologic biopsy analysis poses a significant challenge to clinical trials. Currently, clinical trial endpoints for MASH are mandated to meet one of the following criteria: (i) resolution of MASH without progression to fibrosis or (ii) regression of fibrosis without exacerbation of MASH. Despite the challenges in anti-MASH drug development, the FDA’s approval of the oral small-molecule resmetirom for the treatment of MASH in March 2024 has provided confidence in clinical interventions for MASH patients and has significantly strengthened the advancement of anti-MASH drug development. Given the variety of possible pharmacological therapies, therapeutic options can be comprehensively divided into metabolic, anti-inflammatory, and antifibrotic strategies. In addition, various combination therapies have been extensively explored to optimize therapeutic efficacy while minimizing potential drug-related side effects (Fig. 1; Table 1).

**Table 1.** List of several clinical trial candidate drugs for MASH.

Strategy	Agent	Target	Latest phase	NCT	Latest outcome	Ref
***Nuclear receptor agonists***						
	Resmetriom	THRβ agonist	Approved	NCT04951219 NCT04197479 NCT05500222 NCT03900429	↓ Hepatic steatosis ↓ Liver fibrosis ↓ Plasma LDL-C, TG	[35, 59]
	VK2809	THRβ agonist	Phase II	NCT04173065 NCT02927184	↓ Hepatic steatosis ↓ Plasma LDL-C, TG	[60]
	ASC41	THRβ agonist	Phase II	NCT05462353	↓ Hepatic steatosis ↓ Plasma LDL-C, TG	
	TERN-501	THRβ agonist	Phase II	NCT05415722	↓ Hepatic steatosis	
	Lanifibranor	PPARα/δ/γ agonist	Phase III	NCT04849728 NCT05232071 NCT03459079	↓ Hepatic steatosis ↓ Inflammation ↓ Liver fibrosis	[61]
	Elafibranor	PPARα/δ agonist	Phase III	NCT02704403 NCT03883607 NCT01694849	↓ Liver fibrosis ↓ Inflammation	[62]
	Saroglitazar	PPARα/γ agonist	Phase II	NCT03061721 NCT03863574	↓ Insulin resistance ↓ Hepatic steatosis ↓ Liver fibrosis	[63]
	Pioglitazone	PPARγ agonist	Phase IV	NCT00994682	↓Insulin resistance ↓ Hepatic steatosis ↓ Liver fibrosis	[64, 65]
	Pemafibrate	PPARα agonist	Phase II	NCT05327127 NCT03350165	↓ MRE-based liver stiffness	[66, 67]
	Obeticholic acid	FXR agonist	Phase III	NCT02548351 NCT01265498	↓ Hepatic steatosis ↓ Liver fibrosis ↑ Pruritus	[68, 69]
	Cilofexor	FXR agonist	Phase II	NCT02854605	↓ Hepatic steatosis ↓ Serum bile acids	[70, 71]
	HPG1860	FXR agonist	Phase II	NCT05338034	↓ Liver fat content	
	Tropifexor	FXR agonist	Phase II	NCT02855164 NCT04147195 NCT03517540	↓ Liver fat content	[72]
	TERN-101	FXR agonist	Phase II	NCT04328077	↓ Corrected T1 (cT1)	
	MET409	FXR agonist	Phase II	NCT04702490	↓ Hepatic steatosis	
	CS0159	FXR agonist	Phase II	NCT05591079	No results posted	
***GLP-1R** **agonists***						
	Semaglutide	GLP-1R agonist	Phase III	NCT04822181 NCT02970942	↓ Body weight ↓ Hepatic steatosis	[73]
	Dulaglutide	GLP-1R agonist	Phase IV	NCT03590626	↓ Hepatic steatosis	[74]
	Liraglutide	GLP-1R agonist	Phase II	NCT01237119 NCT02654665	↓ Hepatic steatosis	[75–77]
***Lipogenesis inhibitors***						
	GS-0976	ACC inhibitor	Phase II	NCT02856555	↓ Hepatic steatosis ↑ Plasma TG	[78]
	MK-4074	ACC inhibitor	Phase II	NCT01431521	↓ Hepatic steatosis ↑ Plasma TG	[79]
	Denifanstat	FASN inhibitor	Phase II	NCT04906421	↓ Hepatic steatosis ↓ Inflammation ↓ Liver fibrosis	[80]
	Aramchol	SCD-1 inhibitor	Phase III	NCT04104321	↓ Hepatic steatosis ↓ Plasma ALT and AST	[81]
	Bempedoic acid	ACLY inhibitor	Phase II	NCT06035874	Recruiting	[82]
	BGT-002	ACLY inhibitor	Phase II	CTR20230344	Recruiting	
***Fibroblast** **growth factor** **mimetics***						
	Efruxifermin	FGF21 analogue	Phase III	NCT06215716 NCT06161571 NCT03976401 NCT04767529	↓ Hepatic steatosis ↓ Plasma ALT and AST	[83–85]
	Pegozafermin	FGF21 analogue	Phase II	NCT03486912 NCT03486899 NCT03400163 NCT02413372	↓ Hepatic steatosis	[86, 87]
***Potential of emerging** **therapeutic strategies***						
	MSDC-0602K	MPC inhibitor	Phase II	NCT02784444	↓ Fasting glucose ↓ HbA1c	[88]
	Cenicriviroc	CCR2/CCR5 inhibitor	Phase III	NCT03028740	↓ Inflammation ↓ Liver fibrosis	[89, 90]
	Selonsertib	ASK1 inhibitor	Phase III	NCT03053063 NCT03053050	↓ Inflammation ↓ Fibrosis	[91, 92]
***Combination** **therapy***						
	PF-05221304/PF-06865571	ACC inhibitor/DGAT2 inhibitor	Phase II	NCT04321031 NCT03248882 NCT03776175	↓ Hepatic steatosis (−) Plasma TG	[93]
	Selonsertib/firsocostat/cilofexor	ASK1 inhibitor/ACC inhibitor/FXR agonist	Phase II	NCT02781584	↓ Hepatic steatosis (−) Plasma TG	[70]
	Obeticholic acid/ atorvastatin	FXR agonist/HMGCR inhibitor	Phase II	NCT02633956	(−) LDL-C	[83–85, 94]
	Cilofexor/semaglutide	FXR agonist/GLP-1R agonist	Phase II	NCT03987074	↓ Hepatic steatosis	[71, 86, 87]

### Nuclear receptor agonists

#### THRβ agonists

Thyroid hormones (THs), particularly L-triiodothyronine (T3) and L-thyroxine (T4), serve as central regulators of numerous biological processes [95]. Their physiological effects are exerted by binding to specific nuclear receptors, known as THRα and THRβ, which are widely distributed throughout various tissues [96]. THRβ, a nuclear hormone receptor, is predominantly expressed in the liver, where it functions as an important regulator of metabolic pathways. THRβ agonists can directly modulate gene expression, thereby enhancing FA oxidation (FAO), suppressing DNL, improving insulin sensitivity, and promoting mitochondrial biogenesis [97]. Patients with hypothyroidism often present with abnormal blood lipid profiles, characterized by elevated levels of low-density lipoprotein cholesterol (LDL-C), TGs, and apolipoprotein B (ApoB) [98]. Within the euthyroid population, a decrease in TH levels increases the risk of developing MASLD. Genetic studies have also revealed that people with downregulated THRβ have an increased risk of MASH [97]. This finding validates the rationale for targeting THRβ as a promising therapeutic approach for MASLD. THs have been demonstrated to be effective at reducing liver fat content (LFC) and reversing MASLD in both rodents and humans. However, nonselective activation of THRα may lead to adverse events such as chest discomfort in some patients [99].

Currently, most THRβ agonists are modified and engineered based on the chemical molecular framework of THs. Sobetirome (GC-1), a synthetic analog of TH that is relatively selective for both the binding and activation functions of THRβ over THRα, regulates the expression of high-density lipoprotein (HDL) and LDL receptors (LDLRs) to reduce cholesterol and TG levels [100, 101]. In MASH mouse models, GC-1 has been shown to decrease fat accumulation in the liver. However, due to preclinical adverse events, the development of GC-1 was discontinued [102]. Eprotirome (KB2115) shows high selectivity for THRβ, effectively lowers serum cholesterol and TGs, and shows no cardiotoxicity [103, 104]. However, possible cartilage damage in dogs and hepatotoxicity in humans have hindered its further development [105].

To overcome the effects of THRβ agonists on extrahepatic tissues, “prodrugs” that specifically target the liver have been developed. VK2809 (MB07811) requires oxidation by the cytochrome P450 isoenzyme CYP3A (cytochrome P4503A) to form the active ingredient. In animal models of hypercholesterolemia, VK2809 significantly reduces LDL-C and TG levels with good tolerability [60]. Subsequently, a phase IIb clinical trial for the treatment of MASH was recently initiated (NCT04173065). ASC41 is metabolized by CYP3A4 in the liver and forms an active compound with increased THRβ affinity. Improvements in LDL-C and TG have been demonstrated in a phase I clinical trial, and a phase II clinical trial is currently recruiting patients (NCT05462353). Another prodrug is TERN-501, which can reduce serum cholesterol levels and ameliorate liver steatosis and fibrosis in rodents with hyperlipidemia and MASH. The results from a phase II clinical trial showed that the LFC of MASH patients treated with TERN-501 decreased by 45% at 12 weeks, and both the primary and secondary endpoints were met (NCT05415722).

Resmetirom (MGL-3196), a selective THRβ agonist, effectively targets the liver by exploiting the organic anion-transporting polypeptide 1B1 receptor expressed on hepatocytes [106]. In phase II clinical trial that included 36 weeks of consecutive liver biopsies, patients treated with resmetirom demonstrated a reduction in liver fat over 12 weeks of treatment. Furthermore, exploratory endpoints such as improvements in liver enzymes and tissue fibrosis showed favorable results, with no adverse events related to THRα activity [107]. Resmetirom is currently being tested in four phase III clinical trials, the primary objective of which is to evaluate its therapeutic effects over a period of 52 weeks in biopsy-confirmed patients with MASH. These studies have assessed safety and tolerability using liver biopsies (NCT04197479 and NCT03900429) [35, 59]. During 52 weeks, 966 patients with mild-to-moderate MASH (up to stage F3) received oral treatment with either 80 mg or 100 mg of resmetirom or placebo once daily in a randomized, controlled phase III trial. Compared with 10% of placebo recipients, 25%−30% of resmetirom recipients demonstrated MASH regression without worsening fibrosis, as determined by histologic analysis of biopsy samples. Twenty-five percent of the resmetirom recipients experienced at least one stage of improvement in fibrosis, while the placebo group experienced almost 15% less improvement in the 8-point NAFLD activity score (NAS). The FDA recently approved resmetirom for the treatment of MASH.

#### Peroxisome proliferator-activated receptor (PPAR) agonists

The PPAR family consists of three isotypes: PPARα (encoded by *NR1C1*), PPARδ (synonymous with β, encoded by *NR1C2*), and PPARγ (encoded by *NR1C3*) [108]. Upon ligand binding to the ligand-binding domain of the PPAR protein, PPAR forms a heterodimer with the retinoid X receptor (RXR). The complex binds to specific peroxisome proliferator response elements (PPREs) on DNA, resulting in the transcription of downstream target genes [109]. PPARα and PPARβ/δ-controlled gene expression is associated with mitochondrial and peroxisomal β-oxidative catabolism and ketogenesis [110]. PPARδ regulates the expression of thermogenic genes in adipose tissue and genes involved in FAO in skeletal muscle, white adipose tissue, and the liver. Furthermore, PPARγ plays a crucial role in the regulation of HSCs [111, 112]. However, weight gain is the most important side effect of treatment with PPARγ agonists, e.g., rosiglitazone, due to the increase in fat content in adipose tissue [113].

Multiple PPAR regulators have been identified and used as treatments for MASH [112, 114–116]. Pemafibrate, a novel selective PPARα modulator, has also been used to treat hyperlipidemia [66]. When combined with tofogliflozin, a highly selective sodium-glucose cotransporter-2 (SGLT2) inhibitor, pemafibrate treatment significantly prevents MASH development in rodents [117]. Pemafibrate administration leads to improvements in markers of liver inflammation and fibrosis. Remarkably, patients with lean MASLD respond better to pemafibrate therapy than patients with obese MASLD [67]. The PPARγ agonist pioglitazone reduces hepatic steatosis and inflammation, but only slightly improves fibrosis [64]. It appears that dual or pan-PPAR agonists have better clinical efficacy than single agonists. Oral administration of the dual PPARα/δ agonist elafibranor (also known as GFT-505) reduces steatotic liver inflammation and fibrosis in a mouse model, but its antifibrotic effects were limited in phase II and phase III studies (NCT01694849 and NCT02704403) [62, 118]. Treatment with the PPARα/δ/γ pan-agonist lanifibranor significantly reduces liver steatosis and inflammation in a rodent model of MASH [112, 119]. In a recent phase IIb trial (NCT03008070), lanifibranor treatment significantly resolved MASH without worsening fibrosis [61]. A phase III study evaluating the efficacy and safety of lanifibranor in adult patients with MASH is currently recruiting new patients (NCT04849728). Generally, dual- or multiple-target PPAR agonists could provide greater benefits in the treatment of multiorgan metabolic disorders in MASH. However, balancing the activation effects between the isoforms remains a challenge in the development of PPAR agonists. Based on encouraging results, numerous PPAR agonists have been developed for potential MASH treatment to overcome side effects or increase the efficacy of PPAR activity [120, 121].

#### Farnesoid X receptor (FXR) agonists

Although FXRs were originally found to be nuclear receptors activated by farnesol derivatives [122], subsequent studies showed that FXR is the primary sensor for bile acid (BA) [123, 124]. FXR is predominantly expressed in the liver and intestines, with lower levels in the kidneys, adipose tissue, and adrenal glands [122]. FXR forms a heterodimer with RXR to modulate the expression of target genes [125]. Clinical studies have shown that MASLD is associated with disruptions in BA homeostasis and its related signaling pathways [126]. FXR regulates the transcription of genes involved in the synthesis, absorption, uptake, and transport of BA to maintain BA homeostasis, and in glucose and lipid metabolism [127, 128]. Activation of hepatic FXR increases the expression of small heterodimer partner (SHP), and the FXR-SHP axis inhibits the expression of SREBP and important lipogenic genes [129, 130]. Moreover, FXR activation induces the expression of PPARα [131] and its target genes, as well as the expression and secretion of hepatic fibroblast growth factor 21 (FGF21) [132], both of which are involved in FA transport and oxidation. In addition, FXR controls genes involved in lipoprotein metabolism, including very low-density lipoprotein receptor (*VLDLR*), scavenger receptor B type 1 (*SR-B1*), syndecan-1, ApoCII/III, and fatty acid translocase (*FAT/CD36*) [125]. In summary, FXR activation reduces liver steatosis by suppressing DNL and promoting FFA oxidation. FXR activation can be anti-inflammatory and reduce liver fibrosis [133–135]. Additionally, activation of FXR has been shown to reduce hepatic gluconeogenesis, suppress glycolysis, induce glycogen synthesis, and modulate insulin signaling [127, 136–138].

Consistent with the critical role of FXR in regulating lipid and glucose metabolism in addition to BA synthesis, several FXR activators of endogenous and synthetic ligands have shown potential for clinical applications. Based on their chemical structure, FXR agonists are classified as either steroidal or nonsteroidal. Obeticholic acid (OCA or 6α-ethyl chenodeoxycholic acid; originally known as INT-747) is a semisynthetic steroidal BA derivative that acts as an FXR agonist [139]. OCA is the first FXR agonist approved by the FDA and the first drug considered to be an investigational drug for MASH. A phase III trial (NCT02548351) on OCA is fully enrolled and includes 2477 randomized MASH patients with precirrhotic liver fibrosis, including nearly 1000 who had been taking the study drug for at least four years. In two preliminary 18-month analyses, 25 mg OCA consistently demonstrated a response rate twice as high as that of placebo in reducing liver fibrosis stage, an endpoint consistent with the FDA’s draft guidance. However, the use of OCA has been reported to be associated with side effects such as pruritus and dyslipidemia [68, 69].

Most steroidal BA-like candidates exhibit adverse effects, and thus researchers have focused on developing new selective nonsteroidal FXR agonists. GW4064 was first reported as a non-BA FXR agonist and has been widely used by scientists as a tool to explore the mechanism of action and pharmacology of FXR [140]. Cilofexor/GS-9674, TREN-101, nidufexor, and tropifexor, which were developed based on GW4064, are currently in clinical trials for the treatment of MASH [72, 141, 142]. Phase II trials of HPG1860 (NCT05338034), TERN-101 (NCT04328077), MET409 (NCT04702490), MET642 (NCT04773964), and other nonsteroidal FXR agonists have demonstrated favorable safety profiles and good tolerance with no serious treatment-related adverse events. These compounds significantly reduce liver steatosis and serum BA levels, and improve liver biochemistry in patients with MASH.

FXR agonists have the potential to improve both MASH histology and pathological features of liver fibrosis, as well as excess lipid accumulation, and thus FXR is one of the most promising targets in the field of MASH. However, the side effects of FXR agonists, including hypercholesterolemia and pruritus, still need to be resolved. Exploring novel FXR agonists or multitarget modulators with minimal side effects, along with lower FXR agonist doses in combination with existing or emerging drugs targeting different mechanisms and tailored patient treatments, could reveal the promising future of FXR agonists in MASH therapy.

### Glucagon-like peptide-1 (GLP-1) receptor (GLP-1R) agonists

GLP-1R agonists are incretin-based hypoglycemic agents with therapeutic effects that include appetite reduction, weight loss, and delayed gastric emptying [143, 144]. These agonists also exert hepatoprotective effects through mechanisms such as increasing insulin sensitivity, reducing lipid accumulation, improving liver mitochondrial function, and inhibiting the stress response of the damaged ER. Interestingly, GLP-1 secretion is impaired in MASH patients, which suggests that GLP-1R is an attractive target for the treatment of MASH [145]. Several available GLP-1R agonists (e.g., liraglutide, dulaglutide, and semaglutide) have been shown to clinically alleviate the symptoms of MASH.

Liraglutide is the first once-daily human GLP-1 agonist approved for the treatment of patients with type 2 diabetes mellitus (T2DM) [146]. In the clinical trial NCT01237119, liver biopsy demonstrated resolution of definitive MASH in 39% of patients who received liraglutide for 48 weeks [75]. A Japanese study of MASH patients with glucose intolerance showed that liraglutide along with diet and exercise interventions for six months improved blood biochemistry characteristics of the disease [76]. Additionally, a clinical trial (NCT02654665) revealed that liraglutide was equally effective as a combined diet and exercise program in reducing weight, LFC, and liver damage within six months [77]. Dulaglutide, another long-acting GLP-1R agonist, has been shown to significantly reduce LFC and γ-glutamyl transpeptidase levels in patients with T2DM and MASLD [74]. Another clinical trial is currently evaluating the efficacy of dulaglutide in MASH and diabetic populations randomized to receive either 1.5 mg of dulaglutide in addition to dietary supplementation or dietary supplementation alone for 52 weeks (NCT03648554). Its primary outcome measures include improved histological characterization of MASH by liver biopsy. Semaglutide is another approved antidiabetic GLP-1R agonist and has been demonstrated to lead to the highest percentage of weight loss of any obesity drug to date [147]. In a 72-week double-blind phase II trial (NCT02970942) in patients with biopsy-confirmed MASH and fibrosis, the percentage of resolution of MASH in the semaglutide group was significantly greater than that in the placebo group [73]. A large-scale phase III trial by Novo Nordisk to evaluate the efficacy of weekly subcutaneous injections of semaglutide in patients with noncirrhotic MASH is currently underway (NCT04822181). Its primary endpoints include the resolution of steatohepatitis, improvement in liver fibrosis, and time to first liver-related clinical event.

In addition, emerging evidence suggests that dual GLP-1 and gastric inhibitory polypeptide (GIP) receptor (GIPR) agonists (e.g., tirzepatide), dual GLP-1 and glucagon receptor agonists (e.g., cotadutide), or triple GLP-1/GIP/glucagon receptor agonists (e.g., HM15211) may be more promising than each therapy alone for alleviating MASH and liver fibrosis, but further testing is needed [148]. A 26-week treatment with once-weekly tirzepatide (10 or 15 mg) significantly improved MASH-related biomarkers in patients with T2DM (NCT03131687) [149]. An ongoing phase II trial (NCT05364931) of cotadutide is currently evaluating its safety and efficacy in patients with noncirrhotic MASH with fibrosis. Moreover, another phase II trial (NCT04505436) is currently evaluating the efficacy, safety, and tolerability of HM15211 over 12 months in individuals with biopsy-diagnosed MASH.

### Lipogenesis inhibitors

Excess hepatic DNL leads to TG accumulation and lipotoxicity, leading to hepatic steatosis [26]. The process of FA biosynthesis includes the following steps: glycolysis, lipogenesis, desaturation, elongation, and esterification. The key enzymes involved in the biosynthesis of FAs from carbohydrate substrates include ATP citrate synthase (ACLY), ACC, and FASN [150]. ACLY catalyzes the conversion of cytosolic citrate to acetyl-CoA, which is derived from the tricarboxylic acid (TCA) cycle. ACC induces the carboxylation of acetyl-CoA to malonyl-CoA, and then FASN converts malonyl-CoA to long-chain FAs (LCFAs). The elongation and desaturation of FAs require the elongation of the very long-chain FA protein (ELOVL) and SCD, respectively. SCD catalyzes the synthesis of monounsaturated FAs (MUFAs). In the esterification step, TGs are synthesized by enzymes including monoacylglycerol acyltransferase (MGAT) and diacylglycerol acyltransferase (DGAT). Drugs targeting ACC, FASN, SCD-1, and DGAT2 are currently being advanced into clinical trials for MASH treatment. Recently, ACLY inhibitors and MGAT2 inhibitors have been shown in preclinical studies to have potential for MASH treatment.

#### ACC inhibitors

ACC, a pivotal enzyme in FA metabolism, has attracted considerable attention as a promising therapeutic target for MASH. In mammals, ACC has two isoforms, ACC1 and ACC2. ACC1 is a cytoplasmic protein that catalyzes the conversion of acetyl-CoA to malonyl-CoA, which is further converted into LCFAs by FASN. ACC2 is a mitochondrial protein that regulates FA β-oxidation. Thus, ACC inhibition simultaneously inhibits hepatic DNL and increases FAO [151, 152]. A recent study also showed that ACC inhibition reduces HSC activation. Therefore, ACC inhibition can directly improve liver steatosis, inflammation, and fibrosis [153].

Available ACC enzyme inhibitors, including GS-0976 from Gilead [154], MK-4074 from Merck [79, 155], and PF-05221304 [156], significantly block hepatic lipogenesis in rodents. Furthermore, a novel arachidonic acid 12-lipoxygenase (ALOX12)-ACC1 targeting strategy (a small-molecule intermetatarsal angle 1 (IMA-1)) promotes ACC1 protein degradation and modestly regulates ACC1 activity [157, 158]. Enzyme inhibitors, but not the ACC1 degrader, significantly increase circulating TGs. The use of GS-0976 in the treatment of MASH is in various clinical trial stages. In the clinical trial NCT02876796, GS-0976 reduced hepatic DNL in overweight and obese subjects. In a randomized, double-blind, placebo-controlled phase II trial (NCT02856555), patients with hepatic steatosis (LFC > 8%) were assigned at a 2:2:1 ratio to receive a once-daily oral dosage of 20 mg of GS-0976, 5 mg of GS-0976, or placebo for 12 weeks. The results showed that GS-0976 was safe and well tolerated. Magnetic resonance imaging-based proton density fat fraction (MRI-PDFF) estimated a ≥30% decrease from baseline in 48% of patients who received the high dose and in 23% of patients who received the low dose, both of which differed significantly from the placebo (15%). However, this clinical trial also showed that patients treated with GS-0976 had an increase in plasma TGs [78]. The phase II clinical trial NCT03248882, which tested PF-05221304 (2 mg, 10 mg, 25 mg, or 50 mg), demonstrated a dose-dependent reduction in the LFC from the baseline [93]. The levels of the MASH-related biomarkers CK18-M30 and CK18-M65 were also reduced after the administration of PF-05221304. However, monotherapy with PF-05221304 also increased the serum TG level in a dose-dependent manner.

#### FASN inhibitors

FASN converts acetyl-CoA and malonyl-CoA from simple dietary sugars into palmitate, a saturated FA with 16-carbon atoms. Elevated hepatic palmitate production is deemed a significant initiator of MASH progression [159], thus indicating the potential use of FASN inhibitors as therapeutic agents for MASH [160, 161]. Denifanstat (TVB-2640) is a highly potent selective FASN inhibitor developed by Sagimet Biosciences [161]. In a preclinical study, denifanstat was shown to improve MASH when used as monotherapy or in combination with the GLP-1 agonist semaglutide. In the phase IIa FASCINATE-1 trial (NCT03938246), denifanstat significantly reduced LFC and improved biochemical, inflammatory, and fibrotic biomarkers of MASH after 12 weeks in a dose-dependent manner [80]. In a phase IIb clinical trial (NCT04906421), denifanstat achieved primary and multiple secondary endpoints after 52 weeks of treatment. Administration of denifanstat (50 mg) resolved MASH with a ≥2-point reduction in the NAS (36% in the denifanstat group versus 13% in the placebo group) and improved the ≥2-point reduction in the NAS (52% in the denifanstat group versus 20% in the placebo group). Denifanstat administration improved fibrosis by ≥1 stage without worsening MASH (41% of patients in the denifanstat group versus 18% of patients in the placebo group) (NCT04906421). Recently, Sagimet Biosciences announced plans to conduct a pivotal phase III program to test denifanstat in MASH patients.

#### SCD-1 inhibitors

SCD-1 catalyzes the conversion of saturated FAs into MUFAs. MUFAs serve as substrates for the synthesis of various types of lipids (such as TGs and phospholipids). SCD-1 inhibition reduces TG synthesis [162, 163], and thus SCD-1 inhibitors have been reported to be promising therapeutic agents for the treatment of MASH [164]. Aramchol (icomidocholic acid) is a liver-targeted SCD-1 inhibitor. Aramchol treatment reduced MASH and fibrosis in a mouse model of diet-induced MASH [165, 166]. Administration of aramchol resolved MASH in a phase IIb trial (NCT02279524) (16.7% in the aramchol group versus 5% in the placebo group). In a phase IIb study, fibrosis was reduced without worsening MASH (29.5% in the aramchol group versus 17.5% in the placebo group) [81]. Recently, a phase III trial revealed that 39% of patients showed improvement in fibrosis after at least 48 weeks of aramchol administration, as determined by the MASH Clinical Research Network scoring system (NCT04104321).

#### 3-Hydroxy-3-methylglutaryl-CoA reductase (HMGCR) inhibitors

The elevation of free cholesterol in the liver serves as a distinct marker that differentiates MASH from non-MASH pathology [30]. Given the high occurrence of hypercholesterolemia and cardiovascular disease (CVD), the use of statins, which inhibit HMGCR, has been investigated in patients with MASLD/MASH [167–170]. Moreover, statin treatment halves CVD morbidity and mortality in statin-treated MASLD/MASH patients [171]. However, the results from these studies are difficult to interpret because of the limited patient numbers and short duration of follow-up.

#### ACLY and MGAT2 are possible targets in MASH

ACLY is an enzyme responsible for the formation of acetyl-CoA from citrate in FA and cholesterol synthesis [172]. In hepatocytes, ACLY inhibition reduces lipogenesis, promotes β-oxidation of FAs, and causes cholesterol efflux [173, 174]. Bempedoic acid (ETC-1002) is the first FDA-approved ACLY inhibitor for the treatment of heterozygous familial hypercholesterolemia [175–177]. ACLY silencing or ACLY inhibition alleviates diet-induced MASH and CCl_4_-induced fibrosis in rodents [178, 179]. The effectiveness of bempedoic acid administration for 24 weeks in lowering liver cholesterol in MASLD patients with T2DM is being investigated (NCT06035874). A randomized, double-blind, multiple-dosing, placebo-controlled, phase Ib/IIa study of the novel ACLY inhibitor BGT-002 in MASLD subjects is ongoing (CTR20230344).

MGAT2 catalyzes the production of DG from monoacylglycerol. This important enzyme is highly expressed in human small intestine and liver, and is responsible for the absorption of dietary fat and the synthesis and distribution of TGs [180]. Hepatic *MGAT2* expression is increased in MASLD patients, and knockout of *Mgat2* reduces fat absorption and increases the secretion of GLP-1 and peptide YY (PYY), which are gut hormones that have beneficial effects on blood glucose and appetite remodeling [181, 182]. A recent study showed that the administration of MGAT2 inhibitors reduces liver fibrosis and inflammation in murine models of MASH and reduces body weight in obese adults [183].

### FGF mimetics

#### FGF21 analogs

FGF21 is an atypical member of the FGF superfamily [184]. FGF21 is a secreted hepatokine that circulates to its target tissues (e.g., the liver, adipose tissue, and brain) [185]. FGF21 plays an important role in the regulation of energy balance as well as glucose and lipid homeostasis [186]. Clinical evidence has indicated that FGF21 serum concentrations are elevated in MASLD patients and are positively correlated with intrahepatic TG levels, which suggests that FGF21 may be a biomarker for MASLD and may serve as a key regulator of lipid metabolism in the liver [187–189]. FGF21 has been reported to reduce intrahepatic lipid accumulation by reducing liver DNL and increasing mitochondrial β-oxidation [190].

Furthermore, FGF21 can reduce liver fibrosis, inflammation, and damage by attenuating “multi-hits”, such as oxidative stress, ER stress, and chronic inflammation, in MASLD pathogenesis [191–193]. To overcome the short half-life and poor pharmacokinetics of native FGF21, long-acting FGF21 analogs have been developed and are in various phases of preclinical and clinical research.

Efruxifermin is a differentiated Fc-FGF21 fusion protein designed to mimic the balanced biological activity of native FGF21. This FGF21 analog exhibits balanced receptor binding affinity for the metabolically relevant FGF receptor (FGFR) 1c/2c and 3c [194]. A phase IIa study (NCT03976401) showed that efruxifermin significantly reduces liver fat in patients with MASH and fibrosis stages 1−3 while maintaining a tolerable safety profile [83, 84]. Patients with MASH showed significant regression of fibrosis and resolution of steatohepatitis after 24 weeks of treatment with efruxifermin [85]. In a phase IIb study (NCT05039450), efruxifermin significantly improved the markers of liver injury and fibrosis, and insulin sensitization. An ongoing phase III trial (NCT06215716) is currently evaluating the efficacy of efruxifermin in resolving fibrosis and improving steatohepatitis in patients with precirrhotic MASH, and the SYNCHRONY Real-World trial (NCT06161571) is evaluating the safety and tolerability of efruxifermin in patients with MASH or MASLD. As the first FGF21 analog to enter a phase III trial, efruxifermin, with anti-fibrosis properties, has promising potential for the treatment of MASH.

Pegozafermin, a glycoPEGylated FGF21, is another notable FGF21 analog [86]. Over 24 weeks of treatment demonstrated significant and clinically meaningful improvements in LFC, noninvasive markers of liver fibrosis and inflammation, and lipid markers in MASH patients with stage F2−F3 fibrosis in the ENLIVEN Phase IIb trial (NCT04929483) [87]. In addition, patients with compensated cirrhosis also showed improvements in fibrosis (F4). The data collected at 48 weeks of treatment with pegozafermin showed sustained improvements in liver damage, inflammation, and fibrosis. Several additional phase III trials to further assess the safety and efficacy of pegozafermin in patients with MASH are scheduled for 2024.

#### FGF19 analogs

Apart from FGF21, FGF19 also appears to have beneficial effects on MASH [195]. FGF19 is a hormone that controls the synthesis of BA from cholesterol via CYP7A1. A recent study revealed that FGF19-induced FGFR activation reduces insulin-induced lipogenesis in the liver. Physiologically, FXR activation triggers the secretion of FGF19 in the ileum, which suggests that the induction of *FGF19*, the target gene of FXR, may be an essential factor in the action of FXR agonists. However, studies on its hepatocarcinogenic effects are limited [196, 197]. Recently, a nontumorigenic variant of FGF19, aldafermin (also called NGM282) [198], was studied in MASH patients in a placebo-controlled phase II trial. Aldafermin was found to be well tolerated after 12 weeks of treatment and >70% of patients in the aldafermin group reduced their absolute LFC by at least 5% from baseline, while only 7% of patients in the placebo group achieved the same reduction (NCT02443116) [199, 200]. However, a more recent clinical study revealed that the administration of aldafermin did not significantly improve fibrosis in at least one stage without worsening MASH (NCT03912532) [201].

### Potential of emerging therapeutic strategies

#### Mitochondrial modulators

Mitochondrial dysfunction is closely associated with the progression of simple steatosis to MASH [202]. Targeting mitochondrial dysfunction with mitochondrial pyruvate carrier (MPC) inhibitors and mitochondrial uncouplers is a potential treatment for MASH. In rodents and nonhuman primates, the administration of mitochondrial uncouplers reverses MASLD and MASH [203]. In obese individuals, 2,4-dinitrophenol (DNP), the most well-known mitochondrial uncoupler, is used as a weight-loss drug [204]. To combat deleterious side effects, such as agranulocytosis, hyperthermia, cataracts, and death, several attempts have been made to develop DNP-based pharmacological agents, including DNP-methyl ether (DNPME), controlled-release mitochondrial protonophore (CRMP), DNP loaded LC-gel (DNP-LC gel, an injectable liquid crystal gel), and Compound (6j) [205–208]. These studies suggest that a promising therapeutic approach to improve fatty liver disease is to slightly increase uncoupling and cellular energy expenditure. In individuals with MASLD, MPC may play an important role in the intrahepatic lipid pool, despite minimal flux through this pathway [209]. Pharmacological inhibition of MPC activity improved metabolic parameters, and reduced liver injury and fibrosis [210, 211] [212]. However, in a placebo-controlled phase IIb trial, MPC inhibition by treatment with MSDC-0602K did not significantly improve MASH (NCT02784444) [88].

#### Galectin-3 (Gal-3) inhibitors

Gal-3 is a β-galactoside binding protein associated with many disease processes, including chronic inflammation and fibrogenesis. Gal-3 is highly profibrotic and modulates the activity of fibroblasts and macrophages in chronically inflamed organs [213, 214]. Consequently, Gal-3 inhibitors are being developed as potential treatments for MASH. GR-MD-02 (belapectin), a proposed Gal-3 inhibitor, has been reported to improve histopathological changes in MASH fibrosis in MASH models [215, 216]. However, in a phase II trial in MASH patients with portal hypertension (NCT02462967), belapectin failed to reduce the hepatic venous pressure gradient [217, 218]. Subanalysis revealed that belapectin increased the hepatic venous pressure gradient and prevented the development of esophageal varices in patients without varices at baseline endoscopy. The effectiveness of belapectin in preventing the development of esophageal varices is currently being investigated in a phase IIb/III trial (NCT04365868).

#### CCR2/CCR5 inhibitors

HSC activation and macrophage-mediated inflammation are key events in the development of MASH. In contrast to tissue-resident and self-renewing KCs in the liver, proinflammatory monocyte-derived macrophages (MoMFs) are derived from circulating monocytes that invade the liver upon injury [219, 220]. These cells exhibit CCR2-dependent metabolic, tolerogenic, and homeostatic properties. These MoMFs directly stimulate HSC activation and promote angiogenesis and inflammation. Both HSC and lymphocyte subsets express CCR5, which controls migration and proliferation. CCR2/5 inhibitors, which target disease-promoting liver macrophages and HSCs, have demonstrated efficacy in reducing fibrosis in animal models of MASH and fibrosis, which has led to their advancement to clinical trials. Cenicriviroc, (CVC, also TBR-652 or TAK-652), is a new and potent CCR2/5 receptor antagonist that is taken orally and has shown antifibrotic effects in animal models [221]. CVC significantly improved liver fibrosis compared with placebo in adults with MASH and stage F2 or F3 liver fibrosis in a phase IIb trial (NCT02217475) [89]. A multicenter, randomized, double-blind, placebo-controlled phase III trial (NCT03028740) evaluated the safety and efficacy of CVC. However, the primary efficacy endpoint of determining whether CVC is at least one level more effective than placebo in improving liver fibrosis and preventing the exacerbation of steatohepatitis was not met and, as a result, the study sponsor decided to discontinue the development of CVC [90].

#### Apoptosis signal-regulating kinase 1 (ASK1) inhibitors

ASK1, a member of the mitogen-activated protein kinase (MAPK) kinase kinase (MAP3K) family, controls the p38 MAPK and JNK signaling pathways. In response to a variety of cellular stressors, such as ROS, ER stress, and inflammatory signals, ASK1 is activated through homodimerization and subsequent autophosphorylation. The JNK and p38 MAPK signaling pathways in liver cells can then be activated by phosphorylated ASK1, which leads to apoptosis, the release of inflammatory cytokines, and the induction of fibrogenic genes [222]. Based on these recognized roles of ASK1, ASK1 inhibitors have been developed within the last decade. Selonsertib, also known as GS-4997, is an ATP-competitive ASK1 inhibitor that is currently being evaluated in the treatment of MASH. However, the findings of two phase III trials (NCT03053063 and NCT03053050) of selonsertib were negative [91, 92].

### Combination therapy

Given the complexity of MASH development, combination therapy directed at multiple targets has also attracted interest, as it is expected to yield more benefits and fewer side effects than monotherapy. Theoretically, combinations of drugs with different mechanisms of action against liver steatosis, inflammation, and fibrosis should be considered [223]. A combination of medications can reduce side effects in two ways. Firstly, the combination may allow the use of lower doses to increase tolerability. Secondly, the combination can alleviate the side effects of the first drug. Such combinations should be safe and provide beneficial effects beyond the liver, such as weight loss, insulin sensitivity, and cardiovascular protection.

#### Combination of ACC inhibitors and DGAT2 inhibitors

Current ACC inhibitors, which have already been tested in clinical trials, also lead to a dose-dependent increase in serum TG concentration in patients. Moreover, hypertriglyceridemia significantly increases the risk of CVD in MASH patients. This complication represents a major obstacle to the clinical use of ACC inhibitors for MASH treatment. ACC inhibition results in hypertriglyceridemia, probably due to suppression of polyunsaturated FA (PUFA) synthesis, which in turn results in increased expression of SREBP-1c and glycerol-3-phosphate acyltransferase 1 (GPAT1) and increased very low-density lipoprotein cholesterol (VLDL-C) in the blood [79]. One way to overcome this deficiency is to simultaneously block TG synthesis. DGAT2 is an enzyme that catalyzes the final step in the generation of TGs. Coadministration of the DGAT2 inhibitor PF-06865571 with the ACC inhibitor PF-05221304 reduced LFC and largely eliminated hypertriglyceridemia in a 6-week clinical trial follow-up period (NCT03776175 and NCT04321031) [93]. This trial demonstrated that the coadministration of ACC inhibitors with DGAT2 inhibitors can exert anti-MASH effects while resolving the hypertriglyceridemia associated with ACC inhibition. In addition, hypertriglyceridemia caused by ACC inhibition monotherapy can be reversed by combination treatment with fenofibrate, a PPARα agonist. This combination prevents an increase in plasma TGs and improves LFC and liver biochemistry (NCT02781584) [224].

#### FXR agonists and combination treatments

The most common side effects of FXR agonists in MASH patients are pruritus, increased plasma LDL-C level, and decreased HDL-C level, which may increase the risk of cardiovascular events [225]. Several FXR agonists, including tropifexor, cilofexor, and OCA, are currently being investigated as combination therapies. OCA has been tested in combination with atorvastatin for the treatment of LDL-C elevation. Administration of OCA for four weeks increased plasma LDL-C level, which subsequently decreased when patients also received atorvastatin (NCT02633956) [94]. Cilofexor was tested in combination with firsocostat (an ACC inhibitor) and selonsertib (an ASK1 inhibitor) in the NCT03449446 and NCT02781584 trials [70]. Cilofexor has also been used in combination with the GLP-1 agonist semaglutide and the ACC inhibitor firsocostat. The combination treatment improved liver enzymes, fibrosis, and the NAS on histology and improved liver elastography (NCT03987074) [71].

#### Combination with anti-diabetic drugs

T2DM is an independent risk factor for MASLD progression [226, 227]. Combining anti-diabetic drugs with anti-MASH drugs may help to improve both diabetes-related and liver-related outcomes. The combination of vitamin E, a recommended anti-MASH drug, with pioglitazone, an anti-diabetic drug, resulted in a great response in MASH with T2DM (NCT00063622) [65]. In addition, GLP-1 agonists or SGLT2 inhibitors, which are anti-diabetic drugs intended to be used exclusively for MASH treatment, have been used in combination with anti-MASH drugs. For example, tropifexor is being investigated in combination with the SGLT1/2 inhibitor licogliflozin (NCT03205150) [228]. Cilofexor has been studied in combination with the GLP-1 agonist semaglutide (NCT03987074) [71].

## Summary and perspective

NASH, a term introduced in the 1980s to describe the excessive accumulation of liver lipids in individuals without heavy alcohol consumption, triggers inflammation and cell death [229]. However, advances in pathology have shown that NASH is not only a fatty liver disease but also a metabolic disorder that is closely linked to features of metabolic syndrome such as obesity and T2DM. Therefore, after extensive discussion, the term MASLD/MASH has emerged as a replacement for NAFLD/NASH and includes patients with hepatic steatosis and the presence of at least one of five cardiometabolic risk factors (hypertension, hyperglycemia, overweight/obesity, hypertriglyceridemia, and HDL hypocholesterolemia) [230–232]. In patients with MASLD, regardless of the extent of fibrosis, primary care should focus on weight loss and treatment of cardiometabolic risk factors. This review highlights the complex pathogenesis of the MASH process and summarizes current treatments and potential therapeutic approaches. Regulators of lipid metabolism, e.g., lipogenesis inhibitors and inducers of FAO, immunomodulators, and antifibrotic candidates, as well as their combinations, have been found to be effective.

Clincal medications for MASLD/MASH management include pioglitazone, a PPARγ agonist, and vitamin E. Recently, resmetirom, a selective THRβ agonist, has received unprecedented approval for MASH treatment. Numerous promising therapeutic avenues for MASH suggest the possibility of successful treatment in the future. In recent years, pharmaceutical companies and academic institutions have devoted significant efforts to exploring lipid metabolism, inflammation, and fibrotic signaling pathways to identify novel MASH drugs. As our understanding of the biological mechanisms underlying MASH progression has deepened, we have witnessed both successful and unsuccessful therapeutic strategies, and the development of safe and effective MASH strategies has continued to advance. Despite the clinical failure of several promising drugs, such as the PPARα/δ agonist elafibranor, the FXR agonist OCA, and the ACC inhibitor GS-0976, the search for safer and more effective treatment approaches based on clinical evidence remains steadfast. For example, the pan-PPAR agonist lanifibranor balances the excitatory effects of PPARα/δ/γ and significantly improves fibrosis. Nonsteroidal FXR agonists exhibit promising therapeutic potential with fewer toxic side effects. Additionally, hyperlipidemia induced by ACC inhibitors can be mitigated, to a certain extent, through combination therapies.

The coordinated regulation of FA and cholesterol metabolism, coupled with the reprogramming of mitochondrial function in the liver, such as through THRβ agonists, could represent crucial avenues for treating MASH. The use of multitarget drugs, e.g., biguanide analogs, or drugs with extrahepatic functions (e.g., insulin resistance, adipose tissue dysfunction, and weight loss), has promoted the development of comprehensive MASH treatment. New indications for antidiabetic drugs, e.g., GLP-1R agonists and SGLT2 inhibitors, for MASH warrant further investigation. As MASH embodies extremely complex and heterogeneous mechanisms, personalized combination therapy approaches and clinical combination treatment may offer a greater chance of treating MASH as early as possible. In addition, increasingly sophisticated diagnostic techniques such as histological measurements, and the artificial intelligence (AI)-assisted drug discovery will accelerate the discovery of novel treatments for MASH.

In summary, some challenges also exist even though the FDA recently approved the first drug, and additional studies are needed to better understand the pathogenic mechanisms linking liver metabolism, response to inflammation, and injury in MASLD/MASH development.
